# Vascular injury of immature epiphyses impair stem cell engraftment in cartilage defects

**DOI:** 10.1038/s41598-022-15721-6

**Published:** 2022-07-09

**Authors:** Ali Rashidi, Ashok J. Theruvath, Ching-Hsin Huang, Wei Wu, Elhussein E. Mahmoud, Joe Gerald Jesu Raj, Krzysztof Marycz, Heike E. Daldrup-Link

**Affiliations:** 1 Molecular Imaging Program at Stanford (MIPS), Department of Radiology, School of Medicine, Stanford University, Stanford, CA 94305 USA; 2 Institute for Stem Cell Biology and Regenerative Medicine, School of Medicine, Stanford University, Stanford, CA USA; 3 Department of Surgery, Veterinary School, South Valley University, Qena, Egypt; 4 International Institute of Translational Medicine (MIMT), Malin, Wisznia Mała Poland; 5 Department of Pediatrics, School of Medicine, Stanford University, Stanford, CA USA

**Keywords:** Molecular biology, Stem cells, Diseases, Medical research, Molecular medicine

## Abstract

The purpose of our study was to investigate if vascular injury in immature epiphyses affects cartilage repair outcomes of matrix-associated stem cell implants (MASI). Porcine bone marrow mesenchymal stromal stem cells (BMSCs) suspended in a fibrin glue scaffold were implanted into 24 full-thickness cartilage defects (5 mm ø) of the bilateral distal femur of six Göttingen minipigs (n = 12 defects in 6 knee joints of 3 immature pigs; age 3.5–4 months; n = 12 defects in 6 knee joints of 3 mature control pigs; age, 21–28 months). All pigs underwent magnetic resonance imaging (MRI) at 2, 4, 12 (n = 24 defects), and 24 weeks (n = 12 defects). After the last imaging study, pigs were sacrificed, joints explanted and evaluated with VEGF, H&E, van Gieson, Mallory, and Safranin O stains. Results of mature and immature cartilage groups were compared using the Wilcoxon signed-rank test. Quantitative scores for subchondral edema at 2 weeks were correlated with quantitative scores for cartilage repair (MOCART score and ICRS score) at 12 weeks as well as Pineda scores at end of the study, using linear regression analysis. On serial MRIs, mature joints demonstrated progressive healing of cartilage defects while immature joints demonstrated incomplete healing and damage of the subchondral bone. The MOCART score at 12 weeks was significantly higher for mature joints (79.583 ± 7.216) compared to immature joints (30.416 ± 10.543, p = 0.002). Immature cartilage demonstrated abundant microvessels while mature cartilage did not contain microvessels. Accordingly, cartilage defects in immature joints showed a significantly higher number of disrupted microvessels, subchondral edema, and angiogenesis compared to mature cartilage. Quantitative scores for subchondral edema at 2 weeks were negatively correlated with MOCART scores (r =  − 0.861) and ICRS scores (r =  − 0.901) at 12 weeks and positively correlated with Pineda scores at the end of the study (r = 0.782). Injury of epiphyseal blood vessels in immature joints leads to subchondral bone defects and limits cartilage repair after MASI.

## Introduction

Matrix-associated chondrocyte implants (MACI) and matrix-associated stem cell implants (MASI) have been widely used for the repair of traumatic cartilage defects in animal models^1–3^ and in adult patients^4–6^. However, the value of MACI and MASI for the repair of traumatic cartilage injuries in children has received surprisingly little attention thus far. When compared with their adult counterparts, children have a greater capacity to regenerate small articular cartilage defects^7^. However, relatively large and full-thickness articular cartilage injuries can lead to premature osteoarthritis^8^. Several investigators treated cartilage defects in adolescents and young adults with MACI and found significant functional improvement and pain reduction postoperatively after MACI^9,10^. However, there is limited information about the value of MASI or MACI in younger children, who still have vascularized cartilage. Few investigations of MACI/MASI in immature versus mature animal models thus far lead to inconclusive results. Shimomura et al. reported equal outcomes of synovial mesenchymal stem cell-based cartilage repair in 4- and 12-month-old pigs^11^, while Yamamoto et al. described superior cartilage repair with the application of fibroblast growth factor-2 in 12-weeks old Japanese white rabbits compared to 24-weeks old animals^12^. Immature cartilage is characterized by the presence of vessels while mature cartilage is avascular, as shown in both animal^13,14^ and human^15^ study subjects. We postulated that vascular injury in the cartilage would lead to an ischemic injury of the subchondral bone and negatively impact defect repair.

The Göttingen minipig model is a preferred large animal model for studying stem cell-mediated cartilage repair due to its similar anatomical, biological and biomechanical characteristics compared to human cartilage, including a limited ability to repair full-thickness cartilage defects endogenously^16^ and maturation from vascularized immature cartilage to avascular cartilage^14^. In immature pigs (< 4-month-old), the epiphysis of the distal femur is composed of a small ossification center and about 3–4 mm thick epiphyseal cartilage, which contains an extensive vascular network^14^. The epiphyseal vessels originate either in the perichondral plexus or the underlying metaphysis, crossing the growth plate^17^. Importantly, these epiphyseal vessels branch but do not anastomose^18,19^. During maturation, the epiphyseal vessels slowly regress^14^, resulting in avascular cartilage in adult subjects. This process mimics the cartilage maturation process in humans^20^.

While investigating MASI in Göttingen minipigs, we noticed inferior cartilage repair outcomes in immature versus mature joints. We hypothesized that a cartilage defect in vascularized immature cartilage would interrupt epiphyseal vessels and cause ischemia in the underlying subchondral bone, which would negatively impact defect repair. This is supported by previous reports of subchondral remodeling after chondral injuries in immature pigs^21^. Therefore, the purpose of our study was to investigate, if vascular injury in immature epiphyses affects cartilage repair outcomes of MASI.

## Methods

The administrative panel on laboratory animal care (APLAC) at our institution approved this prospective case–control study (APLAC29859). All applicable institutional and national guidelines for the care and use of large animals, and the recommendations in the Animal Research: Reporting of In Vivo Experiments (ARRIVE) guidelines were followed. Studies were performed in six Göttingen minipigs (Marshall Farms, North Rose, NY) with 24 cartilage defects of the bilateral distal femur, including 12 cartilage defects in three 3.5–4-month-old immature pigs (2 male, 1 female) and 12 cartilage defects in three 21–28-month-old mature pigs (1 male, 2 female). Per recommendation of the APLAC at our institution, we implanted multiple implants per animal in order to minimize the use of large animals for medical research. Our statistical design accounts for a potential dependency of data from multiple implants in the same animal.

### Isolation of mesenchymal stromal cells (MSCs)

Syngeneic mesenchymal stromal cells (MSC) were harvested from the bone marrow of anesthetized Göttingen (Marshall Farms, North Rose, NY) pigs under general inhalation anesthesia with isoflurane according to previously published protocol^22^ (Fluriso, Vetone, MWI, Boise, Idaho; 1–3% in oxygen/1–2 L/min).

#### Bone marrow isolation

A bone marrow aspiration needle (Covidien) was advanced into the anterior superior iliac spine. Approximately 15–20 mL of bone marrow was aspirated into a 30 mL syringe containing 5 mL of heparin anticoagulant. The needle was repositioned several times within the iliac crest to minimize venous blood collection.

#### Cell isolation and maintenance

The marrow aspirate was centrifuged at 1500 RPM for 5 min in a disposable test tube, resulting in 10 mL of cell concentrate, which contained hematopoietic, mesenchymal, and endothelial cells. The cell concentrate was suspended in Dulbecco’s Modified Eagle Medium (DMEM) containing 10% fetal bovine serum (FBS), and 1% antibiotics (penicillin, amphotericin, and streptomycin) (Life Technologies Corporation, NY, USA), and then cultured in CGMP-compliant MSC expansion media (StemPro^®^ group) in 225 cm^2^ cell culture flasks at 5% CO2 and 37 °C. After 5 days of enrichment in MSC selective culture media, the cells were routinely immunophenotyped before transplantation according to the International Society for Cell Therapy (ISCT) requirement^23^ and as previously described^24^.

### Matrix-associated stem cell implantation

Animals were sedated with telazol (2-8 mg i.m./kg), intubated endotracheally, and anesthetized with isoflurane (1–3% in oxygen/1–2 L/min). After surgical disinfection of the knee, a medial patellar skin incision was made, the knee joint was exposed via a lateral dislocation of the patella, and two full-thickness cartilage defects (5 mm ø) per knee were created in the medial femoral condyle, carefully preserving the subchondral endplate, with a biopsy curette (FST, Foster City, CA). The defects were created above and below the midline of the cartilage, 5 mm apart from each other, in the same axis to be visualized simultaneously in the sagittal plane. An independent observer noted the presence or absence of blood oozing from the cartilage defect. The defect was irrigated with sterile saline until no bleeding was apparent. Twelve cartilage defects in six knee joints of immature pigs were implanted with 1 × 10^7^ MSC (n = 12) and secured with fibrin glue (Fibrin and thrombin, Evicel^®^, Etichon, USA). In addition, twelve cartilage defects in three mature control pigs were implanted with 1 × 10^7^ MSC (n = 12) and secured with fibrin glue (Fibrin and thrombin, Evicel^®^, Etichon, USA). Then, the patella was repositioned and the stifle joint capsule, associated muscle layers, and subcutaneous tissue were closed with absorbable suture materials. Finally, the skin was closed with non-absorbable suture materials, the anesthesia was reversed and the animal was returned to its cage, allowing for free movement. We evaluated the joint function in our animals before and at weeks 2, 4, 12, and 24 after the MASI procedure, using a validated locomotion scoring system (www.zinpro.com/lameness/swine/locomotion-scoring). Accordingly, the function and lameness of the animals were scored from 0 to 3: “0” represented easy and comfortable movement with little inducement; “1” represented relatively easy movement but visible signs of lameness in at least one leg; “2” represented lameness in one or more limbs with compensatory behaviors such as dipping the head or arching the back; “3” represented a reluctance to walk and bear weight on one or more legs.

### MRI assessment of cartilage repair

All pigs underwent magnetic resonance imaging (MRI) at 2, 4, 12 (n = 24 defects), and 24 weeks (n = 12 defects). MRIs of both knees were obtained on a clinical 3T MR scanner (Signa HDxt, GE Healthcare), using a receive only knee coil (GE Healthcare) and intermediate weighted proton density (PD) fat saturated (FS) fast spin echo (FSE) sequences (repetition time (TR) = 3700 ms, echo time (TE) = 34 ms, flip angle (FA) = 90°, matrix size = 192 × 192 pixels, field of view (FOV) = 8 cm, slice thickness = 1.3 mm), T2-weighted SE (TR = 3500 ms, TE = 90 ms, FA = 90°, matrix size = 192 × 192 pixels, FOV = 8 cm, slice thickness = 1.3 mm), FS 3D spoiled gradient echo (SPGR) (TR = 50 ms, TE = 7 ms, FA = 30°, matrix size = 192 × 192 pixels, FOV = 8 cm, slice thickness = 1.2 mm) and T2*-weighted gradient echo (GE) (TR = 150 ms, TE = 4.75 ms, FA = 25°, matrix size = 256 × 256 pixels, FOV = 8 cm, slice thickness = 1.3 mm). The total scan time was 62 min. A radiologist (A.J.T., with 5 years of experience in interpreting knee MRI scans) evaluated the subchondral edema and cartilage repair, using OsiriX software (version 10.0, 64 bit; Pixmeo), according to the MR observation of cartilage repair tissue (MOCART) score^25,26^, which assesses nine variables: defect fill, cartilage interface, surface, adhesions, structure, signal intensity, subchondral lamina, subchondral bone and effusion (Supplementary Table S1). The cartilage repair tissue score ranged from 0 (worst) and 100 (best). The subchondral edema was quantitatively scored according to the MR observation of cartilage repair tissue (MOCART) score^25,26^. Accordingly, the subchondral changes were scored from 1 to 4: “1” represented the subchondral bone with no major changes; “2” represented minor edema-like signal in the subchondral bone with a maximum diameter of < 50% of repair tissue diameter; “3” represented severe edema-like signal in the subchondral bone with a maximum diameter of ≥ 50% of repair tissue diameter; “4” represented an osteonecrosis-like signal or subchondral cyst with the longest diameter of ≥ 5 mm.

### Macroscopic, histological, and RT-PCR analysis

The pigs were sacrificed at 12 weeks (n = 3) and 24 weeks (n = 3) for evaluation of long-term cartilage repair outcomes. One experienced investigator who was blinded to the experimental groups scored the macroscopic repair of each cartilage defect based on the scoring system of the international cartilage repair society (ICRS), which evaluates the cartilage defect depth, the integration of the implant into surrounding cartilage, the smoothness of the cartilage surface and the overall defect repair^27^ (Supplementary Table S2). To determine the consistency of the macroscopic evaluation, the same investigator scored the cartilage defect repair according to the scoring system of Pineda et al., which determines the percentage filling of the cartilage defect, the reconstruction level of the osteochondral junction, the visibility of defect margins, and cell morphology^28^ (Supplementary Table S3).

For histological analysis, the knee joints were dissected, femur specimens were placed in 10% buffered formalin for 48 h, washed in deionized water for 4 h, and decalcified for 8 weeks using Cal-Ex™ II Fixative/Decalcifier (Thermo Fisher). After washing in tap water three times for 15 min, the samples were dehydrated in a series of ethanol solutions of increasing concentrations (50%, 60%, 70%, 80%, 90%, 96%) for 15 min at each concentration. The tissues were placed in xylene for 30 min, then transferred to xylene-paraffin solution in a 1:1 ratio for 60 min. Afterward, the samples were incubated in pure liquid paraffin for 24 h at 65 °C. Then the paraffin was changed to the new one for 1 h more, embedded in paraffin, and frozen overnight. The specimens were serially cut into 7 μm fragments^29^ using a microtome (Leica RM2244) and then, the 7 µm tissue fragments were applied to glass slides and stained. The specimens were stained with Hematoxylin & Eosin (H + E), Van Gieson, Mallory, Alcian blue, and Safranin O stains as described previously, and histological grading of the regenerated cartilage was performed on each section according to the Wakitani score^30^ (Supplementary Table S4). The Pineda cartilage repair score was used to evaluate the results of the cartilage regeneration progress^28^. The score ranged from 0 to 14, where a score of 0 indicates normal cartilage architecture, while a score higher than 5 indicates microarchitecture deterioration or more severe cartilage defects. The Pineda and Wakatiani score were evaluated based on the Hematoxylin + Eosin and Alcian blue stainings according to previously published protocols^29,31^. The morphology and architecture of cartilage were evaluated using hematoxylin–eosin staining, and the presence of glycosaminoglycans in the extracellular matrix was determined based on Alcian Blue stains^29,31^. The histological slides were evaluated by two independent pathologists.

For vascular endothelial growth factor (VEGF) Fluorescent staining, mature and immature pig knee tissue sections were blocked with 5% of goat serum (CELLect, cat# 2939149) and 0.1% Tween20 in phosphate-buffered saline for 1 h at room temperature. Subsequently, tissues were stained with primary VEGF monoclonal antibody (clone VG76e, Invitrogen cat#MA1-16626) at 1 in 100 dilutions at 4 °C overnight. Goat anti-mouse Alexa Fluor 488 antibody (Invitrogen, cat#A32723, shown as red color in images for better visualization) was used as a secondary antibody at 1 in 500 dilutions. Fluorescent images were obtained using a Keyence BZ-X710 microscope.

To analyze the VEGF gene expression, after joint dissection, the collected cartilage samples from immature and mature pigs, as well as the control animals (healthy mature pigs), were immediately minced, pulverized using a cryogenic technique, suspended in cooled Trizol (Invitrogen, Carlsbad, and homogenized (Bandelin GM20) as previously described^32^. Next, samples were centrifuged at 12,000×*g* for 5 min at 4 °C to pellet undigested tissue. Cleared supernatant was transferred to a new tube and passed 10 times through a 22G needle. Extruded material was mixed with 200 μL of chloroform by vigorous shaking for 30 s, left to stand for 3 min at room temperature, and then centrifuged at 12,000×*g* for 15 min at 4 °C. Next, the obtained aqueous solution was mixed with sodium chloride and sodium acetate to achieve final concentrations of 1.2 M and 0.8 M, respectively. In order to precipitate the total RNA, 500 μL of isopropanol was added, and the samples were centrifuged at 12,000×*g* for 40 min at 4 °C and washed twice with ethanol. Obtained pellets of total RNA were washed with 70% ethanol and centrifuged at 7500×*g* for 5 min at 4 °C. Then, the RNA pellets were left to dry and finally resuspended in 40 μL of DEPC-treated water. The quantity and quality of obtained RNAs were evaluated spectrophotometrically at 260/280 wavelength (Epoch, Biotek, Bad Friedrichshall, Germany). Digestion of gDNA was performed using PrecisionDNAse Kit (Primerdesign, BLIRT S.A.) and 500 ng of RNA was reverse transcribed with Tetro cDNA Synthesis Kit (Bioline Reagents Limited, London, UK) to synthesize cDNA. The qPCR reaction was performed using SensiFAST SYBR Green^®^ and Fluorescein Kit in CFX Connect Real-Time PCR Detection System (Bio-Rad, Hercules, CA, USA). The reagents for RT-qPCR were derived from Bioline Reagents Ltd. (London, UK). The normalization of targeted transcript levels was performed using the reference genes, i.e., glyceraldehyde-3-phosphate dehydrogenase (Gapdh) and ribosomal 18s RNA. The values of targeted transcripts expression were calculated using the 2–∆∆Ct algorithm in Bio-Rad CFX Maestro Software (Bio-Rad, Hercules, CA, USA). Comparative analysis was made using GraphPad Software (Prism 8.20, CA, USA). All reactions were run in triplicate. Primer sequence information is available upon request.

### Computerized image analysis and histomorphometry

Twelve fields, with an area of 400,000 µm^2^, were randomly selected from each section and were analyzed for the absorbance of each dye including van Gieson, Mallory, and Alcian blue staining. The sections of cartilage images were analyzed with image analysis software (AxioVision Release 4.8.2-SP2 Software, Carl Zeiss Microscopy GmbH, Jena, Germany), which quantifies the level of dye absorbance in the colorimetric count (pixel^2^)/μm^2^. Through a blinded review, two experienced histologists evaluated obtained photographs in accordance with previously published protocol^33^. For histomorphometry, the computerized imaging analytical software (AxioVision Release 4.8.2-SP2 Software, Carl Zeiss Microscopy GmbH, Jena, Germany) was used as previously described^33^.

### Power analysis

The minimal number of animals needed in each experimental group is three, assuming a two-way analysis of variance (ANOVA) for group comparisons with a 0.05 significance level. A two-way ANOVA will have 97% power to detect a variance among the presence or absence of metaphyseal injuries in arthritic joints and will have 91% power to detect differences between immature and mature joints by means of 25% of an SD, assuming that the common standard deviation is 1.000 when the sample size in each group is three. Three animals per group will have 80% power to detect differences of more than 1.5 SD.

### Statistical analysis

The results were presented as mean data with standard deviation (± SD), derived from at least three technical repetitions. Statistical analysis was performed using the Wilcoxon signed-rank test and one-way analysis of variance with Dunnett’s post hoc test. The calculation was made using GraphPad Software (Prism 8.20, San Diego, CA, USA). We correlated quantitative scores for subchondral edema at 2 weeks with quantitative scores for cartilage repair (MOCART score and ICRS score) at 12 weeks and with Pineda scores at the end of the study (at 24 weeks), using linear regression analysis. Furthermore, we correlated quantitative scores for cartilage repair (MOCART score) at 12 weeks with Pineda scores at the end of the study (at 24 weeks), using regression analysis. The healing and locomotion scores were compared with the Wilcoxon signed-rank test. Differences with a probability of p < 0.05 were considered significant.

## Results

### MRI and locomotion scores

**Figure 1:**
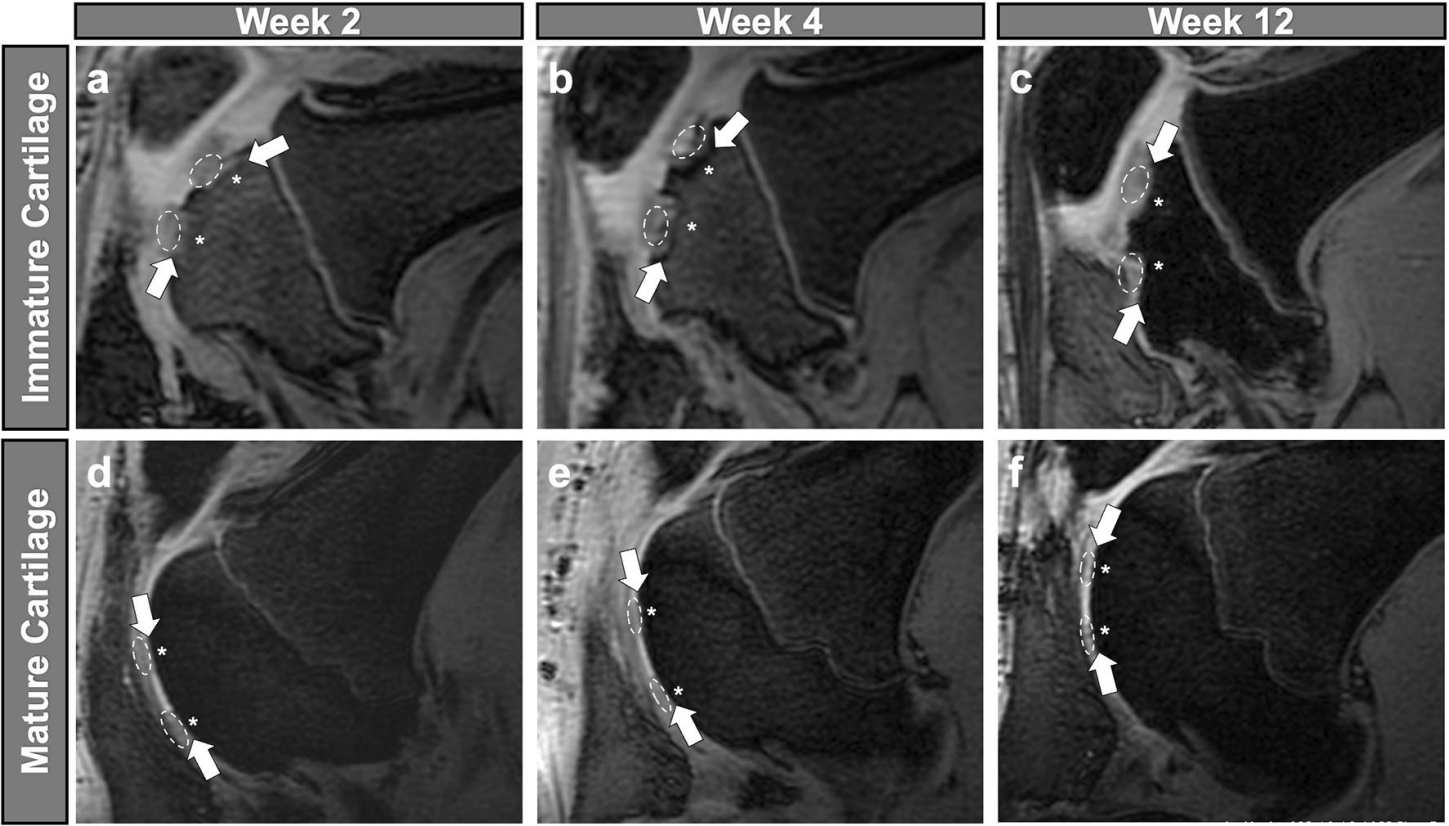
Sagittal SPGR 3D FS knee MRI of the immature and mature pigs after MASI. In the immature knee joint **(a–c)**, at 2 weeks after MASI **(a)**, MRI demonstrates MSC implants (dashed lines) in full-thickness cartilage defects (arrows). On follow-up imaging scans at week 4 **(b)**, the knee joint demonstrates associated subchondral edema (asterisks) and bone defect in the subchondral bone (arrows). The follow-up imaging study of the immature joint at 12 weeks after MASI **(c)** demonstrates a persistent size of the cartilage defect and increasing size of the subchondral bone defect (arrows). In the mature knee joint **(d–f)**, at 2 weeks after MASI **(d)**, MSC implants (dashed lines) in full-thickness cartilage defects and intact subchondral endplate (arrows) are appreciated. In contrast to the immature joint, on follow up MR imaging of the mature joint, the knee joint demonstrates limited subchondral edema (asterisks) and the size of the cartilage defects and subchondral bone defects (arrows) decreases at week 4 **(e)**, and week 12 **(f)** after MASI, consistent with progressive healing.

**Figure 2:**
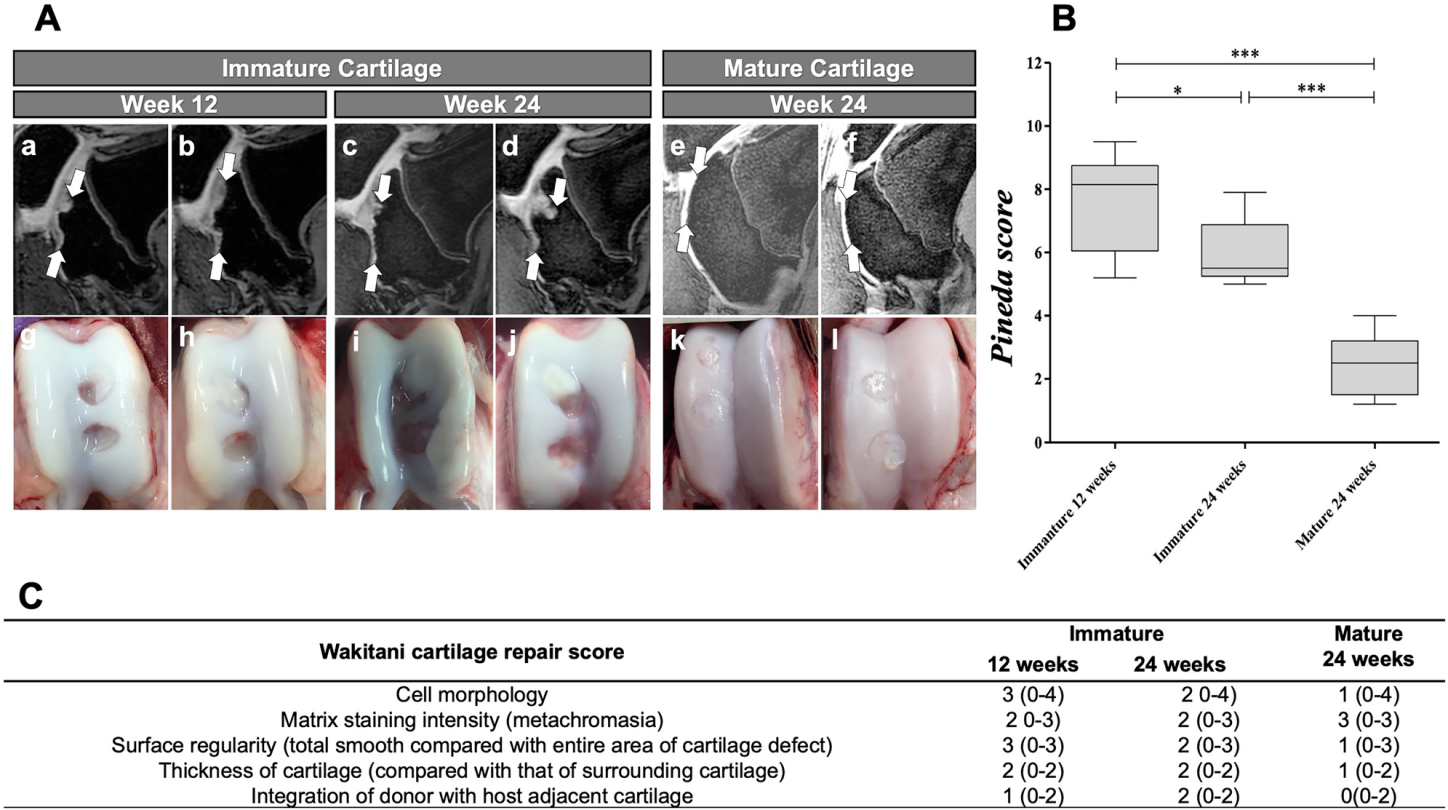
Longitudinal evaluations of cartilage defects in immature and mature cartilage. (**A**) Sagittal SPGR 3D FS knee MRI and macroscopic evaluation of cartilage defects in immature and mature joints at different time points after MASI. **(a,b)** MRI of two representative immature knee joints at 12 weeks after MASI demonstrates persistent cartilage defect which extends into the subchondral bone. **(c,d)** MRI of immature knee joints at 24 weeks after MASI demonstrates further increased cartilage and subchondral bone defects. **(e,f)** By comparison, a cartilage defect in a mature joint has been almost completely repaired at 24 weeks after MASI. There is no subchondral edema or defect. **(g,h)** Macroscopic specimen of the immature knee joints demonstrated incomplete cartilage defect repair at 12 weeks and increasing defects in the cartilage and **(i,j)** subchondral bone at 24 weeks. **(k,l)** By comparison, macroscopic specimens of the mature knee joints demonstrated complete cartilage defect repair at 24 weeks. (**B**) Corresponding Pineda cartilage score of cartilage repair in immature (after 12 and 24 weeks) and mature joints (after 24 weeks). Data are displayed as means and standard deviation of four cartilage defects per group. The Pineda score of cartilage defects of immature joints at 24 weeks was significantly higher compared to the Pineda score of mature joints. (**C**) Corresponding cartilage repair score of immature and mature pigs according to Wakitani cartilage repair scoring system (*p < 0.05, **p < 0.01, ***p < 0.001).

At 2 weeks after MASI, MRI studies showed a bone marrow edema above MSC implants in immature joints and no edema above MSC implants in mature joints. The subchondral endplate was intact in all joints (Fig. 1). At 4, 12, and 24 weeks after MASI, MRI studies of immature joints demonstrated an increasing bone defect in the area of the previous edema. By comparison, no or minimal bone defect was noted in mature joints (Figs. 1, 2A).

**Figure 3:**
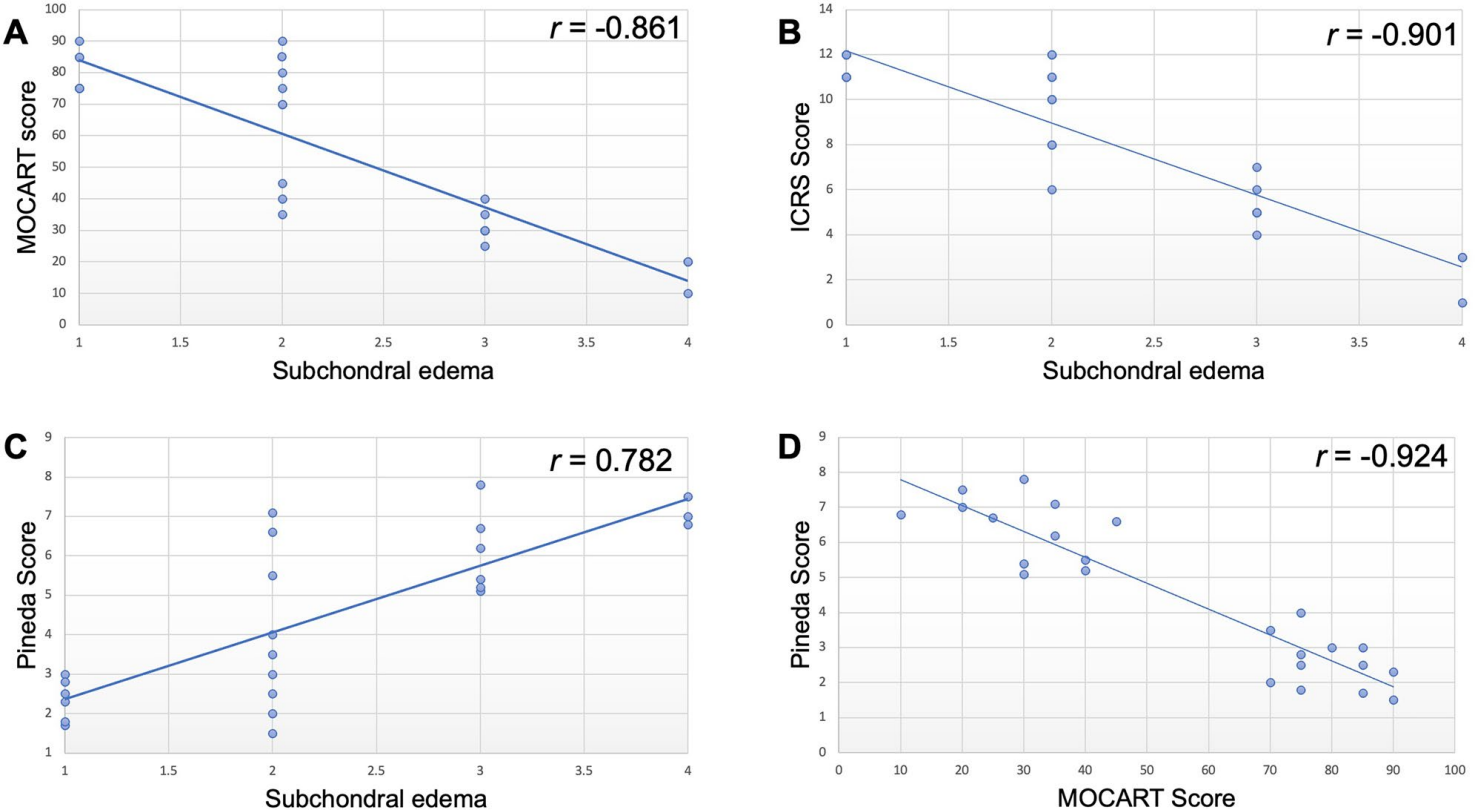
Correlation of quantitative scores for subchondral edema at 2 weeks after MASI with quantitative scores for cartilage repair (MOCART score, (**A**) and ICRS score, (**B**)) at 12 weeks and Pineda scores at 24 weeks (**C**) and correlation of the quantitative scores for Pineda scores at 24 weeks with quantitative scores for cartilage repair (MOCART score) at 12 weeks (**D**).

At 12 weeks, the MOCART score was significantly lower for immature joints (30.416 ± 10.543) compared to mature joints (79.583 ± 7.216, p = 0.002). In order to account for the location of the cell transplants, we compared MOCART scores of defects in the more proximal, retropatellar femur and defects in the more distal femur. We found that the MOCART score was significantly lower for immature joints compared to mature joints for both cell transplants in the superior (37.500 ± 6.892 vs. 80.833 ± 7.359, respectively, p = 0.024) and inferior locations (23.333 ± 8.755 vs. 78.333 ± 7.527, respectively, p = 0.027; Table 1). Similarly, the ICRS score at 12 weeks was significantly lower for immature joints (5.083 ± 2.108) compared to mature joints (11.250 ± 0.753, p = 0.002), irrespective of the defects’ location. Quantitative scores for subchondral edema at 2 weeks were negatively correlated with MOCART scores (r =  − 0.861, Fig. 3A) and with ICRS scores (r =  − 0.901, Fig. 3B) at 12 weeks. Expectedly, the quantitative scores for MOCART and ICRS scores at 12 weeks were positively correlated (r = 0.983).

**Table 1:** MOCART score of superior and inferior defects of immature and mature pigs.

Defect location in femur condyle	Immature pigs	Mature pigs	P-value
Superior	30	85	0.024
	45	70	
	35	80	
	45	90	
	40	85	
	30	75	
Inferior	10	85	0.027
	30	75	
	25	75	
	35	90	
	20	75	
	20	70

The mean locomotion scores were 0.500 ± 0.845 for immature joints, and 0.305 ± 0.624 for mature joints (p = 0.058). At baseline, weeks 4, 12, and 24, the locomotion score was 0 for both immature and mature joints (p > 0.999). In week 1, the locomotion scores were 2.166 ± 0.408 for immature joints, and 1.333 ± 0.816 for mature joints (p = 0.102), and in week 2, the locomotion scores were 0.833 ± 0.408 for immature joints, and 0.500 ± 0.547 for mature joints (p = 0.317).

### Macroscopic, histological, histomorphometry, and RT-PCR analysis

At 12 and 24 weeks, macroscopic evaluations demonstrated incomplete repair of cartilage defects in immature joints and complete repair of cartilage defects in mature joints. In mature joints, the Pineda score (Fig. 2B, p = 0.002), and the Wakitani score (Fig. 2C, p = 0.157) were lower when compared to immature joints. Quantitative scores for subchondral edema at 2 weeks were positively correlated with Pineda scores at 24 weeks (r = 0.782) (Fig. 3C). However, the Pineda scores at 24 weeks were negatively correlated with MOCART scores (r =  − 0.924, Fig. 3D) at 12 weeks.

**Figure 4:**
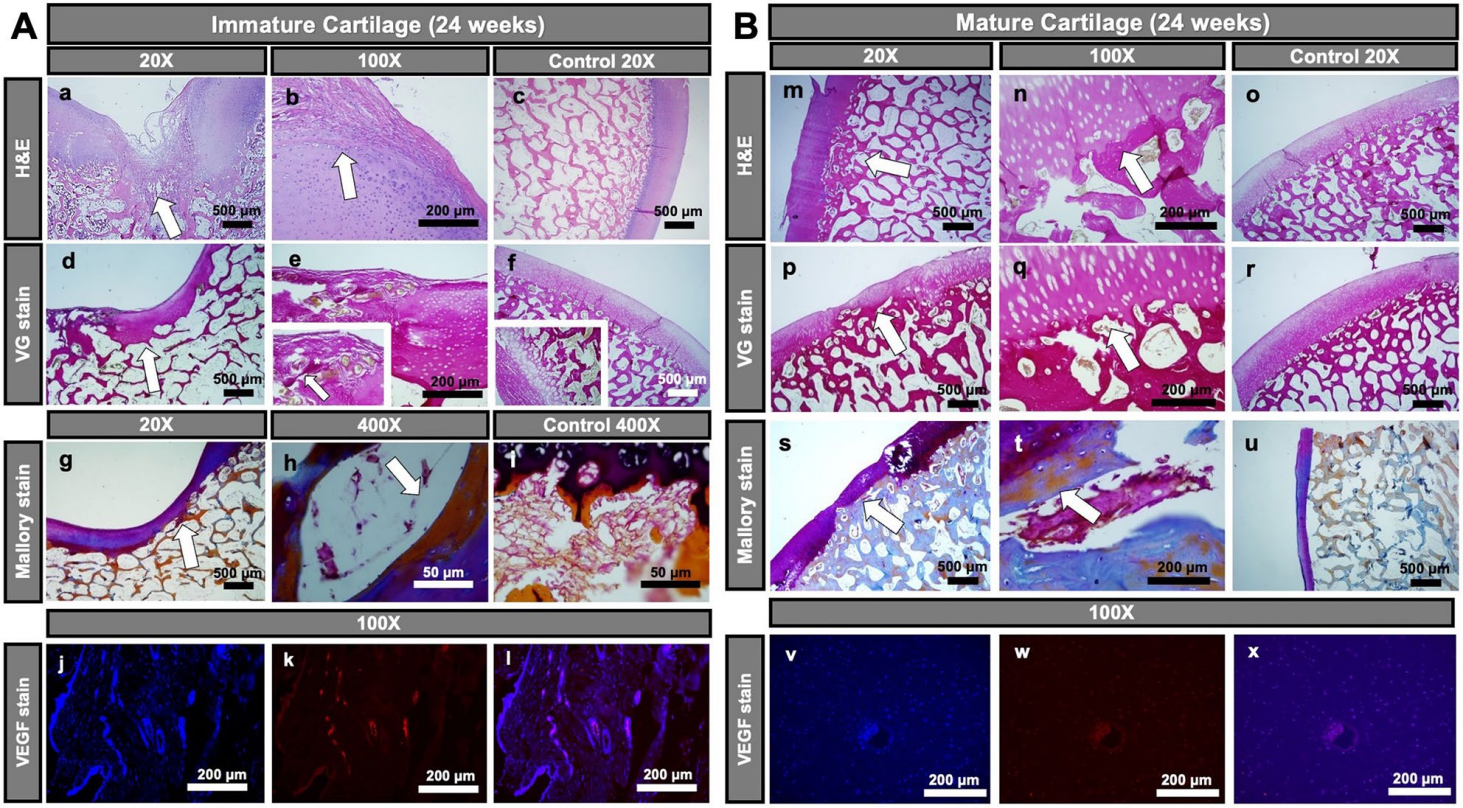
Histological analysis of cartilage repair in immature (**A**) and mature (**B**) pigs at 24 weeks after MASI. (**A**) Immature Cartilage (scale bars: 500 µm, 200 µm, and 50 µm): H&E (**a–c**) and van Giesson’s (**d–f**) staining showed reduced cartilage repair with a thin and irregular surface of the cartilage (**a,b,d,e**) after MASI while proper histological cartilage with dense network of vessels was observed in control samples (**c**,**f**). Significant edema, mononuclear cell infiltration (arrow, **b,e**), and impaired vessels (asterisk, **e**) were observed after MASI. The Mallory stain confirms the presence of elastin and collagen fibers in the calcified part of cartilage (**g–i**) and around the vessels (arrows) indicating reduced cartilage repair after MASI. Fluorescent staining (**j–l**) of cartilage tissue for nucleus (**j**, demonstrated as blue), VEGF (**k**, demonstrated as red), and overlay image (**l**; demonstrated as purple) indicate high VEGF expression and further demonstrate the endothelium of vessels within the immature cartilage. (**B**) Mature Cartilage (scale bars: 500 µm and 200 µm): (**m**) H&E staining demonstrates complete cartilage defect repair with a smooth surface (arrow); (**n**) complete defect repair with proper chondrocyte morphology (arrow); (**o**) a normal control without cartilage defect demonstrates a thin cartilage layer with smooth cartilage surface. (**p**) Van Gieson’s staining demonstrates complete defect repair (arrow); (**q**) complete defect repair with proper chondrocytes morphology (arrow); (**r**) a normal cartilage without defect demonstrates proper chondrocyte morphology. (**s**) Mallory stain demonstrates physiological collagen fiber deposition (arrow); (**t)** Dense network of collagen fiber distribution (arrow) and absence of vessels in cartilage; (**u**) a normal control cartilage with well-developed extracellular matrix. (**v–x**) Fluorescent staining of cartilage tissue for nucleus (**v**, demonstrated as blue), VEGF (**w**, demonstrated as red), and overlay image (**x**; demonstrated as purple) indicate limited VEGF expression.

**Figure 5:**
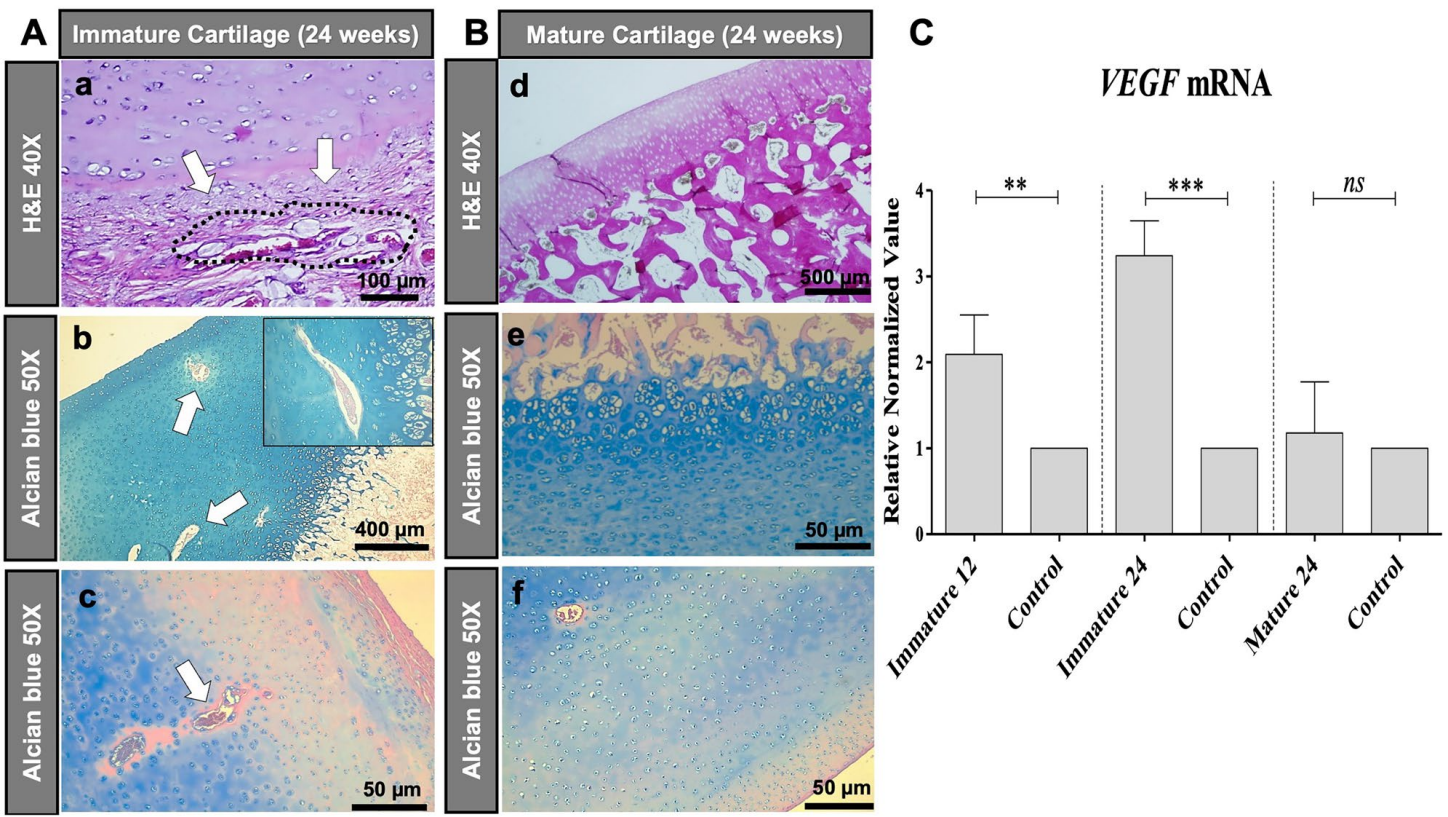
Histological analysis of vessels in the cartilage of immature **(A)** and mature **(B)** pigs at 24 weeks after MASI, and analysis of expression of vascular endothelial growth factor (VEGF) on mRNA level using RT-PCR method **(C)**. (**A**) Immature Cartilage (scale bars: 400 µm, 100 µm, and 50 µm): H&E staining revealed the presence of impaired vessels and mononuclear cell infiltration outside of the vessels (arrows, **a**). Alcian blue staining confirmed the presence of vessels in the immature cartilage (arrows, **b**). In the control samples, vessels with proper structures were observed (arrows, **c**). (**B**) Mature Cartilage (scale bars: 500 µm and 50 µm): H&E (**d**), and Alcian blue stainings (**e**) revealed no vessels in cartilage in mature cartilage after MASI (**d**) as well as in control pigs (**f**). (**C**) The lowest relative expression of VEGF was demonstrated in mature cartilage (Mature 24) when compared to immature (immature 12 and 24) after MASI respectively. Representative data from three independent experiments are shown ± SD (n = 3) (**p < 0.01, ***p < 0.001). *SD* standard deviation, *ns* not significant.

**Figure 6:**
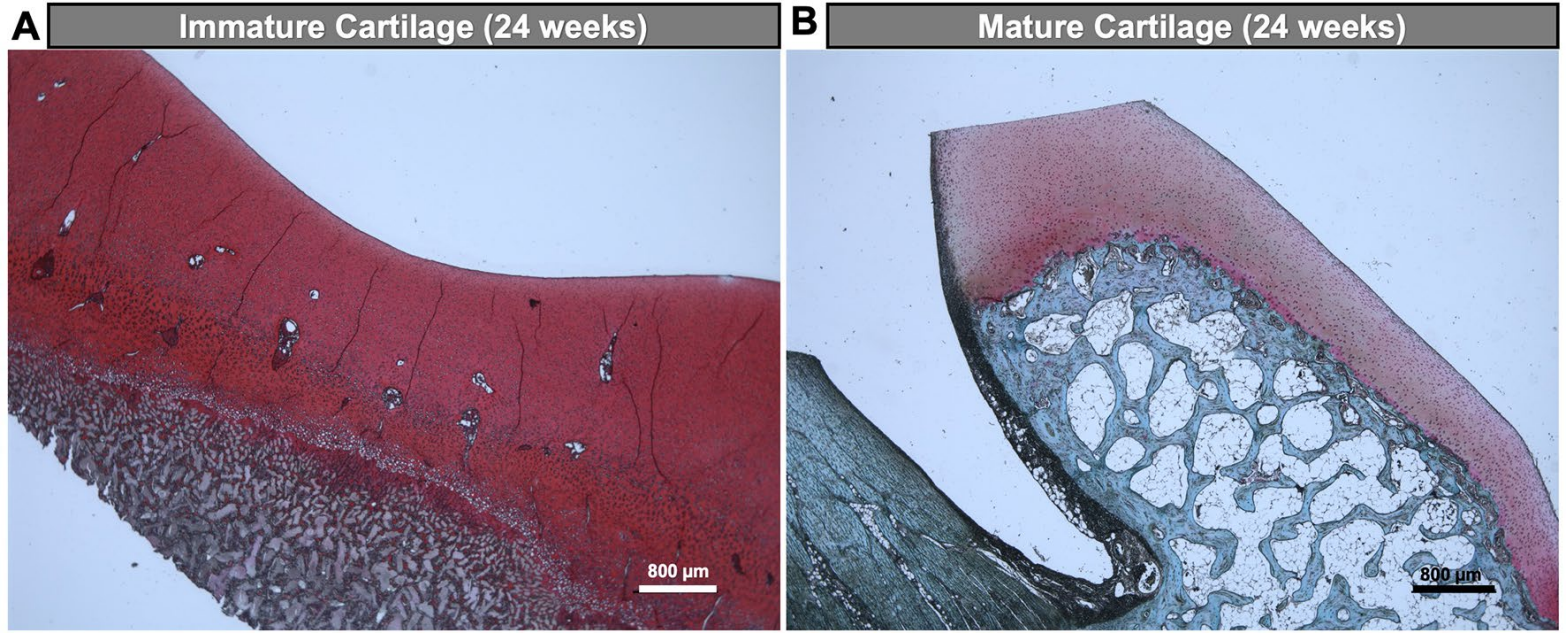
Histological analysis of cartilage repair in immature (**A**) and mature (**B**) pigs at 24 weeks after MASI (scale bars: 800 µm). Safranin O staining demonstrates the presence of microvasculature in the cartilage of immature pigs (**A**) while no vessels were identified in mature pigs’ cartilage (**B**).

**Figure 7:**
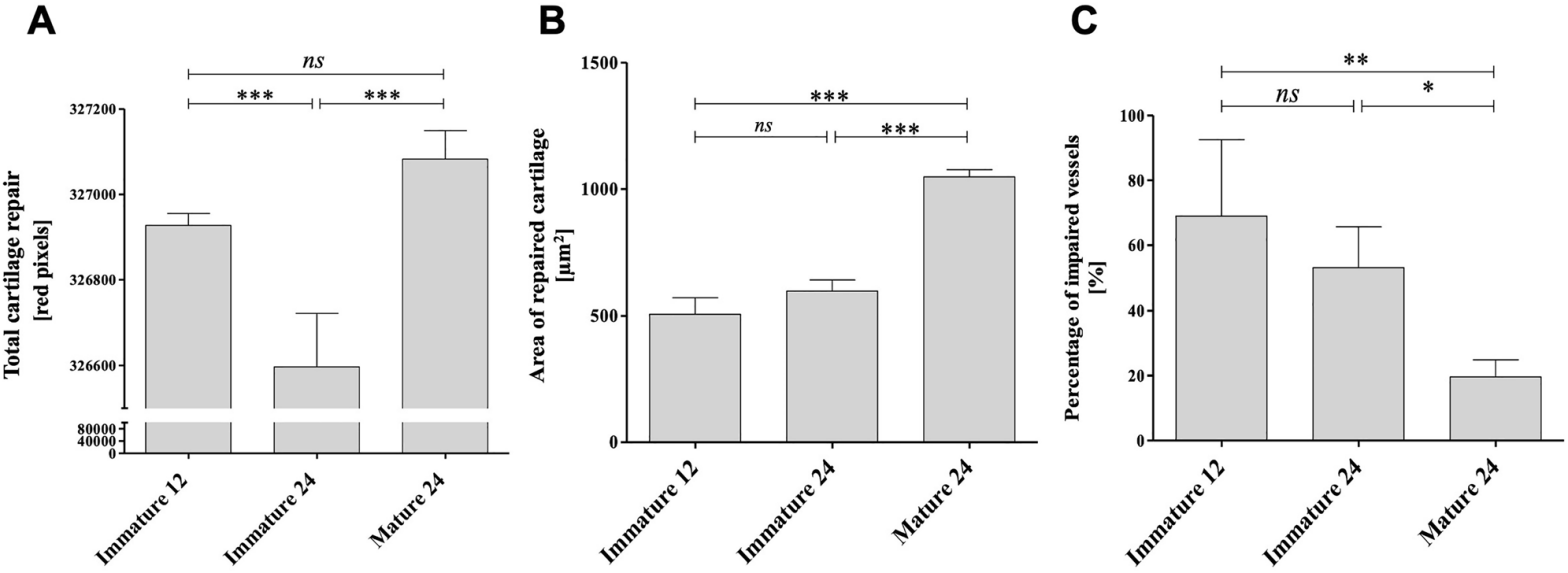
The comparison of total cartilage repair (**A**), the area of repaired cartilage (um^2^) (**B**), and the percentage of impaired vessels (**C**) between immature and mature pigs after MASI. (**A**) The reduced absorbance of van Gieson dye in immature pigs was noted when compared to mature pigs. Reduced dye absorbance of van Gieson, demonstrates a limited cartilage repair and functional deterioration (***p < 0.001). (**B**) The reduced area of repaired cartilage (um^2^) in immature pigs was noted when compared to mature pigs. The lowest area of repair for cartilage defect in immature pigs at 12 and 24 months after MASI indicating on the limited cartilage repair in immature pigs (***p < 0.001). (**C**) Representative data from randomized, twelve fields are shown ± SD (n = 12). An asterisk (*) indicates a comparison between all of the tested groups (*p < 0.05, **p < 0.01). *SD* standard deviation, *ns* not significant.

Histological analysis of the immature joint specimen at 24 weeks after MASI showed disrupted vessels in the cartilage defect and adjacent subchondral bone (Fig. 4Ae). A persistent cartilage defect was noted at 12 weeks and 24 weeks (Fig. 4Aa,b,d,e). H&E stains demonstrated a defect of the subchondral bone with disrupted vessels (Fig. 5Aa–c). The van Gieson and Mallory staining revealed the presence of elastic fibers, elastin, and mononuclear cells in the subchondral defect (Fig. 4Ab,e,g,h). VEGF staining demonstrated abundant microvasculature (Fig. 4Aj–l). The presence of multiple microvessels in immature cartilage was also noted on Safranin O stains (Fig. 6A). The computerized colorimetric analysis of images and histomorphometric investigation revealed the reduced dye absorbance of van Gieson stains, demonstrated a limited cartilage repair and functional deterioration of immature joints at 24 weeks after MASI, and the area with limited cartilage repair when compared to the mature pigs at 24 weeks after MASI (Fig. 7A,B). The histomorphometry analysis revealed the highest percentage of impaired vessels in the immature joint at 24 weeks after MASI indicating reduced cartilage repair progress in comparison to the mature pigs 24 weeks after MASI (Fig. 7C).

By contrast, the histological analysis of mature joints demonstrated no vessels in the cartilage (Fig. 4Bt,v,w,x). The cartilage defect was completely filled with hyaline-like cartilage (Fig. 4Bm,n,p,q). The H&E staining revealed the presence of well-organized chondrocytes (forming columns). No vessels or mononuclear cell infiltration was observed (Fig. 5Bd–f). Van Gieson and Mallory’s stainings confirmed the development of functional cartilage with visible collagen fibers in the injured site and intact blood vessels in the subchondral bone marrow (Fig. 4Bp,q,s,t). VEGF (Fig. 4Bv–x) and Safranin O (Fig. 6B) stainings demonstrated absent microvasculature in mature cartilage. The colorimetric image analysis, as well as histomorphometric investigation, showed the highest absorbance of van Gieson staining in mature pigs after MASI, representing the highest content of collagen as well as the highest rate of cartilage defect repair (Fig. 7A,B). Moreover, the subchondral part of the bone was well integrated with hyaline-like cartilage.

The analysis of gene expression and immunofluorescent staining demonstrated a significantly higher expression of VEGF in immature cartilage compared to mature cartilage (Figs. 4Aj,k,l,Bv–x, 5C).

## Discussion

Our data showed that vascular injury in immature epiphyses affects cartilage repair outcomes of MASI. Cartilage injury in immature joints caused damage to epiphyseal vessels and localized ischemia in the underlying metaphysis, which negatively impacted MASI-mediated cartilage repair.

Fisher et al. observed the development of subchondral bone defects under full-thickness cartilage defects and not partial-thickness defects in the femoral grove of 6-month-old mini pigs^34^. The authors concluded that the development of bone defects under full-thickness cartilage defects was a biological phenomenon and not due to mechanical loading. Our study suggests a vascular injury as the underlying cause of the subchondral bone defects in full-thickness cartilage defects.

In this study, we demonstrated that, in contrast to the mature joint, defects in immature cartilage leads to vascular damage which results in subchondral bone defects and limited cartilage repair. This in turn was strongly correlated with a higher expression of VEGF mRNA. These results are in accordance with findings by Chung and colleagues who demonstrated a critical role of angiogenesis and pro-angiogenic VEGF in the course of cartilage repair in a rat tibial growth plate injury repair model^35^. Previous studies in rat models showed that the healing process of cartilage is mediated by pro-angiogenic factors that modulate chondrocyte precursor differentiation and therefore might constitute a critical role in the cartilage repair process^36,37^. Although the data of Gerber et al. showed that lack of VEGF in growing mice resulted in a greater hypertrophic zone of the growth plate and deterioration of trabecular bone formation in the metaphysis^38^, however, in non-operated animals without cartilage defect.

This study’s results strongly correlate with recent data of Nagao and colleagues, who demonstrated in surgical knee defects in mice (a model of post-traumatic OA in humans), that increased expression of VEGF is associated with catabolic processes in chondrocytes and synovial cells which leads to reduced cartilage repair^39^. Thus, the collective results suggest that impaired vessels with higher VEGF expression in immature cartilage limit cartilage repair and increase the chondral and subchondral defects.

We found that vascular injury in immature cartilage significantly reduced MASI-mediated cartilage repair. The injury to the immature cartilage, which contains microvessels^13,14^, results in an ischemic injury of the underlying bone and a pro-inflammatory environment in the cartilage defect, which leads to oxidative stress and starvation of transplanted cells in the injured cartilage. This is in accordance with previous studies that showed the starvation and catabolic microenvironment results in MSCs death due to progressive apoptosis^40,41^ and various lines of evidence, using cellular models, suggesting the critical role of starvation-induced apoptosis in chondrocytes degradation and cartilage degeneration^42–46^. Our results were not affected by differences in weight bearing or mobility. To limit the stress for experimental animals, we created 5 mm cartilage defects, which do not heal spontaneously, but which also do not cause significant impairment in locomotion. Therefore, the locomotion of the animals was not different in immature and mature pigs.

In young adults with articular cartilage defects, osteochondral autograft transplantation system (OATS)^47^, and autologous chondrocyte implantation (ACI)^48^, are effective surgical treatments. Bentley et al. showed the superior effect of ACI compared with OATS in a 10-year follow-up of young adult and adult patients^49^. Performing ACI using the matrix-associated technique (i.e., matrix-associated autologous chondrocyte implantation, MACI) was introduced as an effective treatment in adolescent patients with chondral defects^10^. Our study proposes that the choice of surgical approach should be informed by the maturity of the underlying defect: A defect in vascularized cartilage will require different approaches than avascularised cartilage.

Limitations of this study include a small number of animals studied. However, we could investigate 24 MASI in cartilage defects of three immature and three mature cartilage, and the results were in accordance with previous studies with larger groups of animals of the same age and strain^2^. While we noted sequelae of vascular injury in cartilage defects on histopathology, we did not apply vascular imaging to our serial MRI studies. In children, vessels in immature cartilage can be well visualized on contrast-enhanced T1-weighted MRI scans^50^. Future vascular imaging studies could further elucidate the relationship between imaging findings of vascular injury and lack of cartilage defect repair.

## Conclusions

In summary, we found that vascular injury in immature epiphyses led to localized ischemia in the underlying metaphysis, subchondral bone defects and limited MASI mediated cartilage repair.

## Supplementary Information

Supplementary Tables.

## Data Availability

All data are published within this manuscript and its Supplementary Information is available upon request. Please contact the corresponding author, Heike E. Daldrup-Link, MD, Ph.D., to request the data from the study.
