# Supplementary material for: Vascular injury of immature epiphyses impair stem cell engraftment in cartilage defects
Source: Sci Rep. 2022 Jul 9;12:11696. doi: 10.1038/s41598-022-15721-6 (PMC9271080; doi:10.1038/s41598-022-15721-6)
Supplement: Supplementary file 1 — Supplementary Tables. [file 41598_2022_15721_MOESM1_ESM.pdf]

Vascular Injury of Immature Epiphyses Impair Stem Cell Engraftment in Cartilage Defects: Investigations in a Large Animal Model

Ali Rashidi[1,2], Ashok J. Theruvath[1,2], Ching-Hsin Huang[1,2], Wei Wu[1,2], Elhussein E. Mahmoud[1,2,3], Joe Gerald Jesu Raj[1,2], Krzysztof Marycz[1,4], Heike E. Daldrup-Link[1,2,5*]

[1]Department of Radiology, Molecular Imaging Program at Stanford (MIPS), School of Medicine, Stanford University, Stanford, CA, USA.
[2]Institute for Stem Cell Biology and Regenerative Medicine, School of Medicine, Stanford University, Stanford, CA, USA.
[3]Department of Surgery, Faculty of Veterinary Medicine, South Valley University, Qena, Egypt.
[4]Department of Experimental Biology, Wroclaw University of Environmental and Life Science, Wroclaw, Poland.
[5]Department of Pediatrics, School of Medicine, Stanford University, Stanford, CA, USA.

*Address correspondence to Heike Daldrup-Link, MD, Ph.D., Department of Radiology, Molecular Imaging Program at Stanford (MIPS), School of Medicine, Stanford University, CA, 94305, USA (email: H.E.Daldrup-Link@stanford.edu).
Tel: +1-650-723-8996

SUPPLEMENTARY TABLES

Supplementary Table S1. MOCART 2.0 Knee Score: Cartilage Repair Tissue Assessment: Grading and Point Scale[1]

			Scoring
1	Volume fill of cartilage defect		
	1	Complete filling OR minor hypertrophy: 100% to 150% filling of total defect volume	20
	2	Major hypertrophy ≥150% OR 75% to 99% filling of total defect volume	15
	3	50% to 74% filling of total defect volume	10
	4	25% to 49% filling of total defect volume	5
	5	<25% filling of total defect volume OR complete delamination in situ	0
2	Integration into adjacent cartilage		
	1	Complete integration	15
	2	Split-like defect at repair tissue and native cartilage interface ≤2 mm	10
	3	Defect at repair tissue and native cartilage interface >2 mm, but <0% of repair tissue length	5
	4	Defect at repair tissue and native cartilage interface ≥50% of repair tissue length	0
3	Surface of the repair tissue		
	1	Surface intact	10
	2	Surface irregular <50% of repair tissue diameter	5
	3	Surface irregular ≥50% of repair tissue diameter	0
4	Structure of the repair tissue		
	1	Homogeneous	10
	2	inhomogeneous	0
5	Signal intensity of the repair tissue		
	1	Normal	15
	2	Minor abnormal—minor hyperintense OR minor hypointense	10
	3	Severely abnormal—almost fluid-like OR close to subchondral plate signal	0
6	Bony defect or bony overgrowth		
	1	No bony defect or bony overgrowth	10
	2	Bony defect: depth < thickness of adjacent cartilage OR overgrowth <50% of adjacent cartilage	5
	3	Bony defect: depth ≥ thickness of adjacent cartilage OR overgrowth ≥50% of adjacent cartilage	0
7	Subchondral changes		
	1	No major subchondral changes	20
	2	Minor edema-like marrow signal—maximum diameter <50% of repair tissue diameter	15
	3	Severe edema-like marrow signal—maximum diameter ≥50% of repair tissue diameter	10
	4	Subchondral cyst ≥5 mm in longest diameter OR osteonecrosis-like signal	0

MOCART: Magnetic Resonance Observation of Cartilage Repair Tissue

Supplementary Table S2. ICRS macroscopic evaluation of cartilage repair[2]

			Points
1	Degree of defect repair		
	1	In level with surrounding cartilage	4
	2	75% repair of defect depth	3
	3	50% repair of defect depth	2
	4	25% repair of defect depth	1
	5	0% repair of defect depth	0
2	Integration to border zone		
	1	Complete integration with surrounding cartilage	4
	2	Demarcating border < 1 mm	3
	3	3/4th of graft integrated, 1/4th with a notable border > 1 mm width	2
	4	1/2 of graft integrated with surrounding cartilage, 1/2 with a notable border > 1 mm	1
	5	From no contact to 1/4th of graft integrated with surrounding cartilage	0
3	Macroscopic appearance		
	1	Intact smooth surface	4
	2	Fibrillated surface	3
	3	Small, scattered fissures or cracs	2
	4	Several, small or few but large fissures	1
	5	Total degeneration of the grafted area	0
4	Overall repair assessment		
	1	Grade I: normal	12
	2	Grade II: nearly normal	11-8
	3	Grade III: abnormal	7-4
	4	Grade IV: severely abnormal	3-1

ICRS: International Cartilage Repair Society

Supplementary Table S3. Pineda score[3]

			Points
1	Filling of defect		
	1	125%	1
	2	100%	0
	3	75%	1
	4	50%	2
	5	25%	3
	6	0%	4
2	Reconstitution of osteochondral junction		
	1	Yes	0
	2	Almost	1
	3	Not close	2
3	Matrix staining		
	1	Intact smooth surface	0
	2	Reduced staining	1
	3	Significantly reduced staining	2
	4	Faint staining	3
	5	No stain	4
4	Cell morphology		
	1	Normal	0
	2	Mostly hyaline and fibrocartilage	1
	3	Mostly fibrocartilage	2
	4	Some fibrocartilage but mostly nonchondrocytic cells	3
	5	Nonchondrocytic cells only	4

Supplementary Table S4. Wakitani score[4]

			Points
1	Cell morphology		
	1	Hyaline cartilage	0
	2	Mostly hyaline cartilage	1
	3	Mostly fibrocartilage	2
	4	Mostly noncartilage	3
	5	Noncartilage only	4
2	Matrix staining (metachromasia)		
	1	Normal (compared to host)	0
	2	Slightly reduced	1
	3	Significantly reduced	2
	4	No metachromatic stain	3
3	Surface regularity		
	1	Smooth (>3/4*)	0
	2	Moderate (1/2 < < 3/4*)	1
	3	Irregular (1/4 < < 1/2*)	2
	4	Severely irregular (l/4 <*)	3
4	Thickness of cartilage		
	1	> 2/3**	0
	2	1/3 < < 2/3**	1
	3	<l/3**	2
5	Integration of donor to host adjacent cartilage		
	1	Both edges integrated	0
	2	One edge integrated	1
	3	Both edges not integrated	2

*Total smooth area of reparative cartilage compared to the whole area of the cartilage defect.
**Average thickness of reparative cartilage compared with that of the surrounding cartilage.

REFERENCES:

1 Schreiner, M. M. *et al.* The MOCART (Magnetic Resonance Observation of Cartilage Repair Tissue) 2.0 Knee Score and Atlas. *Cartilage*, 1947603519865308, doi:10.1177/1947603519865308 (2019).

2 van den Borne, M. P. *et al.* International Cartilage Repair Society (ICRS) and Oswestry macroscopic cartilage evaluation scores validated for use in Autologous Chondrocyte Implantation (ACI) and microfracture. *Osteoarthritis Cartilage* **15**, 1397-1402, doi:10.1016/j.joca.2007.05.005 (2007).

3 Pineda, S., Pollack, A., Stevenson, S., Goldberg, V. & Caplan, A. A semiquantitative scale for histologic grading of articular cartilage repair. *Acta Anat (Basel)* **143**, 335-340, doi:10.1159/000147272 (1992).

4 Wakitani, S. *et al.* Repair of large full-thickness articular cartilage defects with allograft articular chondrocytes embedded in a collagen gel. *Tissue Eng* **4**, 429-444, doi:10.1089/ten.1998.4.429 (1998).
